# Sources of stress and coping strategies among Chinese medical graduate students: a qualitative study

**DOI:** 10.1186/s12909-024-05603-y

**Published:** 2024-06-05

**Authors:** Yanhao Zhang, Xiaoli Lin, Lina Yu, Xue Bai, Xiangyu Li, Wenfei Long

**Affiliations:** 1 Guangzhou university of traditional Chinese medicine, Guangzhou, China; 2 Department of Anesthesiology, The second affiliated hospital of Guangzhou university of traditional Chinese medicine, No. 111 Dade Road, Yuexiu District, Guangzhou, Guangdong China

**Keywords:** Qualitative research, Graduate medical education, Mental health

## Abstract

**Background:**

The incidence of mental health problems among medical graduate students is much higher than among students of other disciplines. This can have adverse consequences for the medical students themselves as well as their future patients. This study aims to understand the pressures faced by Chinese medical students and the current status of mental health education. It also propose recommendations for the current situation and prospects for the future.

**Method:**

The authors conducted in-depth semi-structured interviews with 22 master’s students from five medical schools during November 2023. All interview sessions were recorded and transcribed verbatim. The transcriptions were analyzed using the Colaizzi’s seven-step method.

**Result:**

Three main themes were extracted from the students’ statements: sources of psychological stress, ways to cope with stress, and perspectives on mental health education. The study showed that current mental health education in China is mostly in the form of printed mental health education manuals and mental health lectures, and there is no active tiered intervention for students at different levels. It is suggested that reforms should be made to shift to a model where the school proactively identifies problems and intervenes based on feedback.

**Conclusion:**

This study reveals the widespread psychological stress and shortcomings in current education methods. To address these challenges, institutions should develop tailored interventions, including tiered support systems, open dialogue promotion, and resilience training. Future research should focus on evaluating innovative interventions’ effectiveness, ultimately fostering a supportive environment that enhances students’ success and contributes to a healthier healthcare workforce.

## Introduction

Stress is viewed as a state of real or perceived threat to homeostasis [1]. Chinese medical graduate students face challenges such as longer academic years and high clinical pressures [2]. Research has shown that the overall prevalence of depression among medical students globally is 28.0% [3], with Asian students having a depression rate of approximately 38.0% [4]. In studies conducted in the Chinese national knowledge Infrastructure (CNKI) database, the proportion of students engaging in health-risk behaviors due to stress was as high as 42.33% [5]. The incidence of suicidal ideation among medical students in mainland China is 11.73%, surpassing that of medical students in the United States (11.2%) [6, 7]. However, only 12.9% of depressed medical students who experienced stress and exhibited health-risk behaviors sought treatment [3]. Over the past three years, amidst the global COVID-19 pandemic, the mental health challenges faced by Chinese medical students have become more pronounced [8, 9]. Existing research on medical student stress primarily focuses on cross-sectional surveys of student anxiety, depression, and related psychological abnormalities, with relatively little research on how medical students cope with stress and alleviate resulting health issues. Current research on stress among medical students mainly focuses on cross-sectional surveys of anxiety, depression, and related psychological abnormalities. Unfortunately, there is limited research on how medical students can cope with stress and alleviate the resulting health issues caused by stress. As future clinical practitioners, medical postgraduates under stress may potentially engage in substance abuse and increase the risk of irreversible harm, such as medical errors [10, 11]. Urgent attention from educational institutions, society, and the government is needed to address the mental health of Chinese medical students. Additionally, scholars have emphasized the importance of enhancing medical students’ psychological resilience and strength.

Moreover, enhancing the psychological resilience and strength of medical students is particularly crucial. Some researchers have improved students’ stress resistance through methods such as autonomous training, progressive muscle relaxation, and Mindfulness-Based Stress Reduction (MBSR) [12–17]. These experiences may shed light on the direction of mental health education for Chinese medical students. Therefore, this study aims to propose an initiative to increase awareness of mental health education for medical graduate students by understanding the various pressures they face, mechanisms for reducing stress, and the acceptance of mental health education. It also suggests providing personalized psychological adjustment methods for this population and designs references for interventions to improve graduate students’ mental health for society, government, and universities.

## Method

The present study revolves around the formulation of interview guidelines based on positive psychology theories. Educational psychology is the scientific study of the fundamental principles governing teaching and learning within educational environments [18]. Positive psychology primarily aims to stimulate and strengthen individual reality and latent capabilities, leading to the development of positive personality traits [19]. These traits, in turn, facilitate individuals in adopting more effective coping strategies. We conducted semi-structured interviews with 22 medical postgraduate students of Chinese nationality who were enrolled in medical or related programs at six universities across three countries. Data collection, organization, and analysis were completed between November and December 2023. All study materials were reviewed by the Ethics Committee of Guangdong Provincial Hospital of Chinese Medicine, and all participants provided informed consent.

### The researchers and participants

We formed a qualitative research team consisting of six members. The team included a university professor specializing in medical education, two doctors engaged in clinical psychology work, and three research postgraduate students (two in nursing and one in psychology). Initially, we extensively reviewed relevant literature on positive psychology theories to develop a draft of the semi-structured interview guidelines. Two clinical doctors conducted preliminary interviews using the draft guidelines with four eligible research postgraduate students, making revisions to the guidelines. The revised guidelines were then handed over to the university professor for further modifications, leading to the final version. Three research postgraduate students were responsible for data collection and organization, while the analysis stage was a collaborative effort involving the entire research team.

We employed convenience sampling to recruit participants for this study, ensuring that there would be a good rapport between interviewers and interviewees, enabling open and honest expression of their thoughts and feelings. The inclusion criteria for participants were as follows: (1) enrollment in a medical graduate program at a university or a comprehensive university with a medical major; (2) good communication skills, and clear verbal expression, absence of mental disorders; (3) informed consent and voluntary participation in the study. Exclusion criteria included individuals who had already graduated, taken a leave of absence, dropped out, or failed mid-term assessments. Out of the 22 interviewees, 20 were enrolled in Chinese universities, while two were studying in foreign universities. Their ages ranged from 22 to 27 years old, with nine being male and 13 being female. The participants were pursuing master’s degrees, and 15 (68.2%) came from middle-income families. Please refer to Tables 1 and 2 for specific details.

**Table 1:** Overview of participants

No.	Gender	Age	Grade	Relationship status	Finances	Major
***N1***	Male	23	2	Single	FS/M	Nursing
***N2***	Female	24	1	Single	FS/L	Clin med
***N3***	Female	22	1	Single	FS/M	Clin med
***N4***	Female	25	2	In love	FS/M	Anesthesiology
***N5***	Female	23	1	Single	FS/H	Clin med
***N6***	Female	23	2	In love	FS/H	Clin med
***N7***	Male	23	2	Single	FS/M	Nursing
***N8***	Female	23	1	Single	FS/M	Nursing
***N9***	Female	23	1	Single	FS/M	Anesthesiology
***N10***	Female	26	1	In love	HS/M	Anesthesiology
***N11***	Female	23	1	Single	FS/M	Clin med
***N12***	Female	26	2	In love	FS/M	Nursing
***N13***	Female	24	1	Single	FS/H	Clin med
***N14***	Female	29	1	In love	HS/M	Nursing
***N15***	Male	23	1	In love	FS/M	Nursing
***N16***	Male	24	2	In love	FS/M	Anesthesiology
***N17***	Male	26	3	In love	FS/M	Nursing
***N18***	Female	25	DG	In love	FS/L	Nursing
***N19***	Male	26	3	In love	FS/M	Clin med
***N20***	Male	28	DG	In love	FS/M	Nursing
***N21***	Male	27	3	Single	FS/M	Anesthesiology
***N22***	Male	28	DG	Single	FS/H	Clin med

**Table 2:** Overview of participants

Gender			
Male	9	40.91%	
Female	13	59.10%	
**Age**			
21–23	9	40.91%	
24–25	5	22.73%	
26–27	4	18.18%	
28–29	3	13.64%	
**Major**			
Clinical medicine	8	36.36%	
Anesthesiology	5	22.73%	
Nursing	9	40.91%	
**Type of Master degree**			
Academic	9	40.91%	
Professional	13	59.10%	
**Grade**			
First	10	45.45%	
Second	6	27.27%	
Third	3	13.64%	
Delay	3	13.64	
Family financial situation*			
Low-income	3	13.6%	
Middle-income	15	68.2%	
High-income	4	18.2%	

* Low income: annual household income less than 60,000 rmb (8388 US dollar); Middle-income: annual household income more than 60,000 rmb (8388 US dollar); High-income: annual household income more than 1.2 million rmb (167,753 US dollar)

### Data collection

Interviews were conducted with eligible participants using a pre-established semi-structured interview guide. Prior to the interviews, we explained to the participants the purpose and methodology of the study, assuring them that their privacy would be respected, and their personal information would not be disclosed. Participant numbers instead of real names were used during the interviews. We also followed the principle of convenience for the participants, agreeing on suitable interview times and locations to ensure a confidential, quiet, and undisturbed environment throughout the interviews.

During the interviews, we obtained informed consent from the participants, and if any doubts or concerns were raised, we immediately halted the interview. We fully respected the participants’ willingness to express themselves and refrained from evaluating the viewpoints they presented. Instead, we enriched the overall research process by using timely probes and follow-up questions. In order to capture accurate information, we recorded the conversations using two recording devices, while carefully observing the participants’ facial expressions and emotional changes, making authentic records of the interview process.

### Interview guide

1. Brief introduction of yourself.
2. A detailed description of your current state, including mental and physical ones.
3. Description of sources of stress in life and ways to relieve them.
4. Perspectives on approaching the future about study and career.
5. Perspectives on mental health education. The forms of receiving mental health education and desired future of receiving mental health education.

### Data analysis

The interview will be transcribed into a computer file within 24 h after the interview is concluded. Then, the interviewee and one researcher within the team will manually verify and reorganize the transcribed data to ensure that the interviewee’s statements are not misunderstood or distorted. Two graduate students will then use the Colaizzi [20, 21] 7-step analysis method to analyze the reorganized interview data: (1) Read the data repeatedly to fully understand the interviewee’s statements; (2) Identify meaningful statements word by word; (3) Encode recurring viewpoints; (4) Collect codes to form common concepts. After completing these four steps, the two graduate students will exchange opinions and accept or delete the formed codes and common concepts. In cases of significant disagreement, a third graduate student will join to make a joint decision in order to minimize the biases caused by the analysts’ subjective intentions. (5) Elaborate on the common concepts and incorporate typical descriptions provided by the interviewee. After completing the fifth step, the elaborated concepts and the interviewee’s typical descriptions will be reviewed by professors from relevant medical schools within the research team to eliminate the narrow analysis resulting from the analysts being graduate students themselves. (6) Construct themes; (7) Provide the obtained themes to the interviewee to ensure the authenticity and accuracy of the results.

## Result

According to the interviewee’s statements, three themes emerged:

1. Sources of stress for medical graduate students: The interviewees highlighted various factors that contribute to their stress levels during their studies.
2. Ways to alleviate stress for medical graduate students: The interviewees shared helpful approaches they employ to manage and reduce stress in their lives.
3. The importance and necessity of mental health education. The interviewees emphasized the importance of mental health education in medical graduate programs to support the well-being of students.

To maintain anonymity, each interviewee was assigned a unique number instead of disclosing their identities.

### Sources of stress for medical graduate students

Medical graduate students face pressure from multiple sources. According to the interviews, they reported increased pressure compared to their previous stages, such as undergraduate studies or work. Additionally, different groups of students, based on academic years and admission methods, experienced varying levels of stress, suggesting different layers of pressure.

#### Stress from economic concerns

The pressures faced by medical graduate students primarily stem from economic concerns. These pressures can be attributed to two key factors. Firstly, medical students experience a prolonged duration of study compared to their peers, resulting in a delayed achievement of financial independence. While their same-aged counterparts may have already established themselves economically, medical students remain dependent on financial support. This discrepancy in financial status creates significant stress for medical graduate students. Secondly, the economic pressure experienced by medical students is further influenced by their family’s financial situation. For those from financially constrained backgrounds, the burden of financial responsibilities and expectations can be particularly overwhelming. Balancing academic demands with financial obligations adds to the already demanding nature of medical education.

N2: Compared to my peers, they have already achieved basic financial independence and no longer rely on their parents for living expenses… As my parents get older, I feel more pressure than before.

N11: There will be financial pressure, and I really want to be able to earn my own money because many of my friends have started working and earning salaries, which makes me anxious.

N22: I haven’t graduated yet, and I estimate that it may take me another year to graduate, which means I will be close to 30 by then. The (financial) pressure is quite high, and most of my peers have already bought cars, houses, and gotten married, while my graduation seems to be far off.

#### Stress from academic studies

Academic pressures also significantly impact medical students, with sources of stress varying between academic years. In lower academic years, students may experience stress due to a lack of clarity and direction regarding their research projects. This ambiguity can lead to uncertainty and anxiety about their future career path. In contrast, middle and upper academic years are faced with the pressure of producing and publishing research papers. The successful completion of these papers is vital for their academic progress and can significantly impact their career prospects.

N15: During the graduate stage, there are more things to consider compared to the undergraduate stage. It involves writing research papers, and in terms of research, one is just beginning to delve into it and there is a lot that one doesn’t understand. This can lead to feelings of anxiety. (Lower academic years)

N6: Can’t help but think too much and do too little when it comes to writing research papers and the pressure to continue pursuing a PhD in the future. (Middle academic years)

N20: The current pressure is to quickly meet the graduation requirements and revise my thesis. (Delayed graduate student)

#### Stress from interpersonal relationships

In addition to the factors mentioned earlier, interpersonal relationships play a significant role in putting pressure on medical students, especially in their interactions with supervisors, peers, and clinical preceptors. The dynamics in these relationships can contribute to higher stress levels. One specific group that faces intensified interpersonal pressure is individuals transitioning from clinical practice to an academic setting. Compared to their counterparts with no prior clinical experience, these individuals often encounter more challenges in navigating interpersonal relationships. The increased pressure can stem from various sources, with the loss of personal privacy being a prominent factor. In the learning environment, which requires close collaboration, feedback, and evaluation, personal boundaries are breached, leading to feelings of vulnerability and heightened stress.

N9: I feel like there is a hierarchical relationship with my advisor, so I am afraid to communicate with them, fearing that I might say something wrong and disappoint them. (About advisor)

N12: It feels like a job, with a superior-subordinate dynamic, where I have to say what they want to hear, even if it means telling lies constantly. (About advisor)

N5: It’s lonely being a graduate student. Even when my roommate is in the dorm, we don’t have much in common, and I feel a strong sense of distance between people. (About classmate)

N17: It’s difficult to develop a close relationship with my roommate. When I return to the dorm, it feels like I’m invisible. I find a secluded corner, sit down, and live in my own world (About classmate).

N4: I don’t like the atmosphere in the department, and the management style of the head nurse is suffocating (About clinical mentor).

N18: When nursing graduate students are in clinical practice, the level of expertise and technical skills of the clinical instructors is inadequate. Some of the theoretical lectures provided by the instructors cannot keep up with the pace of progress in nursing and may be outdated. (About clinical mentor)

The career development of medical graduate students is a topic of great concern and pressure. They often face the decision of choosing between working in clinical settings, pursuing further academic studies, or taking on non-clinical positions related to medicine. Our research indicates that nursing graduate students in particular show some aversion towards clinical work. This aversion may stem from concerns about emotional burnout and job-related stress. However, it is important to recognize that these concerns are not unique to nursing students but are likely shared by medical students in different disciplines. The findings highlight the need to support medical students in making informed career decisions, while also prioritizing their personal well-being and self-care. Moreover, promoting the exploration of non-clinical opportunities and expanding the scope of medical training can offer valuable alternatives for medical students. The COVID-19 pandemic has significantly impacted medical students, with school and hospital lockdowns and restrictions generating substantial pressure. The implementation of measures such as social distancing, personal protective equipment protocols, and limitations on clinical placements can cause significant disruptions to medical education and clinical training. However, it is noteworthy that the pandemic has also increased public recognition and appreciation for healthcare workers, including medical students. This recognition has the potential to improve medical students’ sense of professional identity and increase their sense of purpose. The outpouring of support and recognition strengthens medical students’ connection to their career choice, motivating them to persevere despite the associated challenges.

### Strategies for medical graduate students to cope with stress

Medical students often turn to exercise to relieve stress and express their emotions, while seeking very few alternative methods. However, the current stress relief options available to them are limited, focusing predominantly on extroverted emotional release. Interestingly, during interviews, none of the respondents mentioned the utilization of introverted strategies to enhance their psychological resilience and inner strength.

N1: (Ways to relieve stress) Talking to the advisor and exchanging ideas with senior colleagues in research. Additionally, relaxing activities like going back to the playground for a run or listening to music and watching dramas.

N3: Facing the problem directly, even if not following the planned schedule, gradually completing tasks. I also accept feedback from friends, reflect on myself, and might adopt their perspectives.

N6: Venting out emotions. There must be an outlet, not keeping everything inside.

N21: Currently, there may not be many ways to alleviate the situation, so it’s essential to face the stress head-on. Dealing with graduation-related issues can indeed be urgent.

### Mental health education of medical students

Medical students recognize the significance of mental health education for themselves as well as for diverse social groups and communities. Currently, psychological education for medical graduate students is primarily delivered through online campaigns, offline lectures, and distribution of psychological health handbooks. However, both in terms of format and significance, it has not met expectations.

N1: With the development of society, every individual faces numerous psychological health issues. Therefore, I believe that psychological health education is highly necessary.

N11: Not only graduate students, but individuals from various social strata and age groups all require it. Psychological health education is highly necessary.

N2: Currently, mental health education is primarily conducted in classrooms. The information is diverse, and when we enroll or before classes, we are shown short films on mental health education and provided with brochures on the topic.

N4: I prefer a more entertaining form of education, with more activities. I enjoy interactive formats as they tend to be more engaging and fun, rather than just lectures.

N6: Mental health education should not be about someone telling me what to do, but rather me sharing my thoughts with them. They can then identify the conflicts in my mind and help resolve my troubles. Ultimately, it would be beneficial to have personalized guidance tailored to each individual’s specific concerns.

Currently, mental health education for medical students tends to be formalistic and does not effectively address their mental health issues. However, medical graduate students are eager to receive psychological education that allows them to actively participate and acquire practical skills to cope with these issues. They prefer a more interactive approach rather than just lectures.

N6: It is indeed very formalistic. Even if you express your concerns, they won’t solve them. They may not even be aware of the issues students are facing… When responsible teachers come to our dormitory, it feels like a leadership inspection, just going through the motions, very formalistic. Mental health education should not be about someone telling me what to do, but rather me sharing my thoughts with them. They can then identify the conflicts in my mind and help resolve my troubles. Ultimately, it would be beneficial to have personalized guidance tailored to each individual’s specific concerns.

N14: I personally would not go to those psychological counseling offices set up for students. If I have psychological issues, I would rather seek help elsewhere. I feel like it’s just for show.

N18: I don’t like the form of lectures or public accounts. They just copy some PPTs and course materials online, which are useless, and students don’t pay attention to them at all.

N4: I prefer a more entertaining form of education, with more activities. I enjoy interactive formats as they tend to be more engaging and fun, rather than just lectures.

## Discussion

### Source of stress and ways students cope with

The essence of the research, conducted through semi-structured interviews, reveals that medical graduate students face various common sources of stress. Initially, transitioning from undergraduate to graduate status induces a sense of discomfort. Heinen’s study identifies that stress among medical students in their first year correlates with personal resources and emotions [22]. Furthermore, delayed financial independence compared to peers is a stressor, as most students rely solely on family support due to the demanding clinical and research workload [23]. This mirrors the situation in many Western countries. In Su’s study, it was found that a good supervisor-graduate student relationship can enhance the positive impact of psychological capital on graduate students’ professional commitment [24].

Meanwhile, this study explored the current methods employed by medical graduate students to alleviate stress. Exercise is one common approach; it allows medical students to release inner emotions and improves both physical and mental well-being, consequently enhancing their daily academic and professional performance. Research indicates a dose-response relationship between lack of exercise and adverse mental health outcomes, including self-harm and suicide attempts, highlighting the necessity of promoting physical activity among university students [25]. Additionally, music serves as a common relaxation method for students. Linnemanna’s research found that music can alleviate daily stress for students, aligning with the positive direction advocated by positive psychology theories [26]. However, due to long working hours, these coping mechanisms cannot always be guaranteed, and the study suggests that only a minority can strengthen inner psychological resilience and strength through these strategies.

Psychological education is one of the crucial measures to promote the mental health of medical graduate students [27], primarily achieved through: (1) curriculum education; (2) regular lectures and workshops by experts or experienced physicians; (3) distribution of mental health materials both online and offline; (4) resources such as online videos, applications, etc.; (5) personalized guidance tailored to individual personalities and needs [28]. Currently, psychological education for medical graduate students in China mainly consists of online activities, offline lectures, and distribution of mental health handbooks. Most students hold a negative attitude towards the methods and approaches employed in school psychological education, perceiving it as primarily formalistic during the graduate stage, with limited practical significance. Limited by the fact that funding for Chinese universities and research institutions mainly comes from national financial subsidies, attention and resources for students’ mental health tend to be overshadowed by support for research and clinical work, making personalized guidance even more challenging. In fact, in the interaction between mentors and students, the relationship tends to be more hierarchical rather than educational and guiding, neglecting the role and influence teachers should have. As the primary person responsible for students’ academic and personal development, mentors’ neglect and students’ resulting self-isolation and reluctance to communicate lead to a sense of disconnection between both parties, depriving students of a means to build a healthy psychological environment. Research indicates that whether mentors provide appropriate support can significantly impact students’ academic output [29], potentially related to the incentives and pressures mentors face in their positions. Due to the heavy academic workload and limited free time, graduate students receive fewer resources for mental health assistance, contributing to the persistent and severe mental health issues among medical graduate students in China.

### Current approaches to supporting students’ mental education

The psychological state is a constantly changing process, and relying solely on a single psychological test at the beginning of the school year to measure students’ psychological state throughout the entire learning period is insufficient. Schools must increase their focus on students’ mental health and consider it as important as academic performance. We should conduct extensive research to identify the differences in issues faced by graduate students at different stages and use this information to determine timely psychological interventions at different time points.

The results of this study indicate that academic pressure, financial stress, and interpersonal relationship pressure are common stressors among medical graduate students. For academic pressure, pre-entry education is necessary to help students transition from undergraduates to graduates and avoid confusion about their learning lives. Therefore, recommendations for introductory professional books and career development literature can be made during graduate admissions, along with introductions to upcoming course plans and schedules. Additionally, under the background of the “Internet Plus” era, early online meetings and collaborations with senior students can also help newcomers integrate into the new environment [30].

The study found that most graduate students rely on family financial support. Although China has implemented a standardized training system for physicians, graduate students are in a blind spot of the system and currently cannot receive standardized training rewards, even though these rewards can only ensure basic survival for doctors. Therefore, it is necessary to improve the working conditions of medical graduate students [31]. In this study, interpersonal relationship stress mainly comes from the relationship between graduate students and their mentors. Mentors should pay more attention to students’ mental health rather than just their academic performance and work. The graduate stage should be a process where teachers and students jointly research scientific problems [32], rather than just focusing on outcomes, which may lead to teachers only caring about students’ research results and neglecting the cultivation process.

There are clear differences in mental health issues among graduate students. Tailored psychological education for different groups based on their characteristics is the key to addressing the current psychological health education for medical graduate students, rather than mere formalism. For special groups, such as those returning to campus from clinical work, assigning them to the same dormitory to unify their schedules as much as possible could be beneficial. Although assigning separate dormitories can address privacy concerns, it may not be fair to other graduate students. The premise of solving the problem is to minimize the emergence of new problems. For students who delay graduation, we could assign them a new graduation advisor to reduce their psychological pressure effectively.

### Future measures to enhance students’ mental education

Research has found that psychological issues among graduate students, particularly in the field of medicine, are significant social concerns. Despite the challenges posed by academic, financial, and social pressures, these pressures are regarded as essential aspects of personal development, shaped by societal realities. However, external measures can only rectify issues after they arise, making it imperative for graduate students to cultivate strong resilience and coping mechanisms to mitigate negative emotions. Thus, enhancing proactive self-adaptation abilities among graduate students is crucial for stress alleviation [33]. Moreover, prioritizing prevention over treatment as graduate students’ psychological issues evolve into mental illnesses underscores the importance of establishing robust mechanisms for early detection and intervention within educational institutions, supported by governmental policies and funding. Educational institutions should provide scientifically effective intervention models, including the implementation of positive tiered interventions based on continuous feedback loops, fostering a culture of open dialogue and support for mental well-being. Integrating psychological education courses and practical resilience-building activities into existing curricula is essential. On the other hand, increasing parents’ understanding of graduate student mental health issues is essential [34], enabling guardians to recognize the pressures faced by graduate student groups, especially in identifying abnormal psychological and behavioral changes in their children, which is also one of the important forces for early detection and prevention of graduate student mental health issues.

Building upon these recommendations, society should actively assume corresponding responsibilities. Under government guidance, strengthening collaboration between universities and social institutions to establish a mental health counseling service system in society is crucial [35]. Establishing a diverse collaborative network is a complex and winding process. Establishing a comprehensive medical graduate student psychological education system within universities requires understanding and tolerance from families, concrete actions from schools, and strong support from government and society.

### Limitations

Although this study focuses on current medical master’s students, their perspectives, as learners, may not fully consider the practical feasibility within the real-world context. In subsequent research, we look forward to incorporating insights from educators involved in medical master’s student mental health education to ensure maximal safeguarding of students’ mental well-being within existing conditions and without compromising the well-being of other demographics.

Several strategies for addressing mental health challenges among medical master’s students are proposed in this study. Unfortunately, apart from enhancing students’ self-psychological resilience, other recommendations tend towards macro-level interventions. These findings require extensive empirical validation and cooperation from all stakeholders. In subsequent research, we aspire to adopt more specific measures, as only a plethora of testable ideas can gradually enrich the discourse surrounding mental health education for medical master’s students.

## Conclusion

This study underscores the urgent need for comprehensive mental health support among medical graduate students in Chinese campuses. The results reveal the prevalence of psychological stress within this group and the inadequacies of current mental health education methods. Themes such as identified sources of stress, students’ coping strategies, and perspectives on mental health education provide valuable insights for addressing these challenges. Looking forward, it is recommended that educational institutions develop proactive mental health interventions tailored to the diverse needs of medical students. This includes establishing positive tiered interventions based on continuous feedback loops, fostering open dialogue, and promoting a culture supportive of mental health. Additionally, integrating practical coping skills training and resilience-building activities into the curriculum can enable students to more effectively manage internal stressors. Furthermore, future research efforts should focus on evaluating the effectiveness of innovative mental health interventions, such as peer support programs and online mental health resources. Longitudinal studies tracking the mental health outcomes of medical students can offer valuable insights into the sustained impact of intervention strategies. By prioritizing mental health education and implementing evidence-based interventions, we can create a more supportive and resilient environment for medical graduate students, ultimately enhancing their academic success and professional development. This not only benefits individual students but also contributes to cultivating a healthier and more effective workforce in healthcare.

## Data Availability

The data that support the findings of this study are available from the authors upon reasonable request and with the permission of authors.

## Abbreviations

CNKI: Chinese national knowledge infrastructure PPT Power Point Microsoft
